# Fam70A binds Wnt5a to regulate meiosis and quality of mouse oocytes

**DOI:** 10.1111/cpr.12825

**Published:** 2020-05-11

**Authors:** Na‐Na Zhang, Teng Zhang, Wen‐Yi Gao, Xin Wang, Zi‐Bin Wang, Jin‐Yang Cai, Yang Ma, Cong‐Rong Li, Xi‐Chen Chen, Wen‐Tao Zeng, Fan Hu, Jian‐Min Li, Zhi‐Xia Yang, Chun‐Xiang Zhou, Dong Zhang

**Affiliations:** ^1^ State Key Lab of Reproductive Medicine Nanjing Medical University Nanjing Jiangsu China; ^2^ State Key Lab of Stem Cell and Reproductive Biology Institute of Zoology Chinese Academy of Sciences Beijing China; ^3^ Analysis and Test Center Nanjing Medical University Nanjing Jiangsu P.R. China; ^4^ The Second Hospital of Changzhou Nanjing Medical University Changzhou Jiangsu China; ^5^ Animal Core Facility Nanjing Medical University Nanjing Jiangsu P.R. China; ^6^ Drum Tower Hospital Medical College of Nanjing University Nanjing Jiangsu China

**Keywords:** Fam70A, meiosis, mouse, oocytes, Wnt5a

## Abstract

**Objectives:**

Little is known about the roles of integral membrane proteins beyond channels, carriers or receptors in meiotic oocytes. The transmembrane protein Fam70A was previously identified as a likely “female fertility factor” in Fox3a‐knockout mouse ovaries where almost all follicles underwent synchronous activation and the mice became infertile very early. However, whether Fam70A functions in oocyte meiosis remains unknown. Therefore, the present study aimed to address this question.

**Materials and Methods:**

Co‐immunoprecipitation, immunogold labelling‐electron microscopy, co‐localization and yeast two‐hybrid assays were used to verify the interaction. Antibody or small interfering RNA transfection was used to deplete the proteins. Immunofluorescence, immunohistochemistry and live tracker staining were used to examine the localization or characterize phenotypes. Western blot was used to examine the protein level.

**Results:**

Fam70A was enriched in oocyte membranes important for normal meiosis. Fam70A depletion remarkably disrupted spindle assembly, chromosome congression and first polar body extrusion, which subsequently increased aneuploidy and abnormal fertilization. Moreover, Fam70A directly bound Wnt5a, the most abundant Wnt member within oocytes. Depletion of either Fam70A or Wnt5a remarkably increased adenomatous polyposis coli (APC), which stabilizes active β‐catenin and microtubules. Consequently, depletion of either Fam70A or Wnt5a remarkably increased p‐β‐catenin (inactive form) and acetylated tubulin, while APC knockdown remarkably decreased these two. Furthermore, Fam70A depletion remarkably reduced Akt phosphorylation.

**Conclusions:**

Fam70A regulates meiosis and quality of mouse oocytes through both canonical and non‐canonical Wnt5a signalling pathways.

**Abbreviations**
APC: adenomatous polyposis coli
ATP: adenosine triphosphate
DMSO: dimethyl sulfoxide
EDTA: Ethylene Diamine Tetraacetic Acid
EGTA: Ethylene Glycol Tetraacetic Acid
Fam70A: family with sequence similarity 70, member A
GCs: granulosa cells
GV: germinal vesicle
GVBD: germinal vesicle breakdown
HEPES: N‐2‐hydroxyethylpiperazine‐N‐2‐ethane sulfonic acid
IgG: Immunoglobulin G
IP: immunoprecipitate
IP‐MALDI: immunoprecipitation and Matrix‐assisted laser desorption ionization
IVF: in vitro fertilization
IVM: in vitro maturation
LC3B: microtubule‐associated protein 1 light chain 3 beta
MALDI‐TOF‐MS (simplified as MALDI): matrix‐assisted laser desorption/ionization time of flight mass spectrometry
MI: metaphase I
MII: metaphase II
MT: microtubule
mTOR: mammalian target of rapamycin
NLR: nucleotide‐binding domain and leucine‐rich repeat‐containing
PHEM: the buffer with 60 mM PIPES 25 mM HEPES 10 mM EGTA 2 mM MgCl and pH 6.9
PIPES: 1,4‐Piperazinediethanesulfonic acid
PN: pronucleus
PND: post‐natal day
RT‐PCR: reverse transcription‐polymerase chain reaction
SCMC: subcortical maternal complex
SDS‐PAGE: Sodium dodecylsulphate polyacrylamide gel electrophoresis
Wnt5a: wingless‐type MMTV integration site family, member 5A

## 1 INTRODUCTION

Compared with somatic cells, meiotic oocytes are unique in several aspects. In addition to differences in cell division (meiosis vs mitosis), various maternal proteins exist in other tissues at low level whereas are uniquely or predominantly expressed and function within oocytes. For example, NLR family pyrin domain containing 5 (Nlrp5), also known as Mater protein homologue, belongs to the Nlrp family and is enriched within ovaries and oocytes. Nlrp5 is a subunit of the subcortical maternal complex that regulates cytoplasmic lattice formation, organelle positioning and distribution.^1^ Nlrp5 depletion results in precocious sister chromatid separation and chromosome misalignment due to a reduction in centromere cohesion.^2^ Notably, the Nlrp5 level is remarkably decreased in oocytes of ageing mice.^2^ Furthermore, Nlrp5 is crucial for normal mitochondrial and endoplasmic reticulum distribution.^3^ and calcium homoeostasis.^4^ GDF9 and BMP15 are two well‐known oocyte‐predominant maternal proteins that are important for oocyte maturation.^5^, ^6^ However, a limited number of studies have investigated the functions of oocyte‐predominant maternal proteins during meiosis.

Moreover, although integral membrane proteins account for about 30% of the total proteome, little is known about whether or how these proteins function during meiosis, except for those functioning as channels, carriers or receptors. Notably, a transcriptome‐wide screening in Fox3a‐knockout mouse ovaries identified 348 likely "female fertility factors," among which 13 are integral membrane proteins.^7^ Fox3a is a well‐known transcription factor downstream of the mTOR pathway, and it represses the expression of genes required for follicle activation and development. In Fox3a knockout mouse ovaries, almost all follicles undergo synchronous activation and the mice became infertile very early (4‐month‐old).^7^ Therefore, genes with increased expression following Fox3a knockout may be important for follicle activation and development.

The Fam70 family includes two integral membrane proteins, Fam70A (gene ID, 245386) and Fam70B (gene ID, 272465), both of which have a conserved Fam70 domain in addition to several transmembrane motifs. To the best of our knowledge, no functional reports on these proteins are currently available. Based on expression data from the National Center for Biotechnology Information (NCBI), Fam70A is predominantly expressed in human and mouse ovaries, compared with other tissues, while Fam70B is not present in human ovaries and is expressed at low levels in mouse ovaries (Figure S1A‐D). We verified that Fam70A was more abundant in mouse ovaries than Fam70B (Figure S1E). Furthermore, Fam70A but not Fam70B was screened as a "female fertility factor," suggesting that it serves a unique role in ovaries and oocytes.^7^

The present study revealed that Fam70A directly bound an oocyte‐predominant Wnt family member, Wnt5a, to regulate oocyte meiosis through the canonical Wnt‐β‐catenin pathway, which is a highly conserved signalling pathway important in a number of physiological processes, including early embryonic development, organ formation and tissue regeneration.^8^, ^9^, ^10^ Furthermore, Fam70 was shown to regulate the activity of Akt, a well‐known meiosis regulator.^11^, ^12^, ^13^

## 2 MATERIALS AND METHODS

### 2.1 General chemicals, reagents and animals

Chemicals and reagents were obtained from Millipore Sigma unless otherwise stated. Institute of Cancer Research (ICR) mice (female, age 3‐4 weeks) used in this study were supplied by Vital River Experimental Animal Technical Co. All animal experiments were approved by the Animal Care and Use Committee of Nanjing Medical University and were performed in accordance with institutional guidelines.

### 2.2 Antibodies

The following primary Abs were used: Rabbit anti‐Fam70a Ab (Cat#: bs‐11006R; Bioss); Rabbit anti‐Wnt5a Ab (Cat#: A12744; ABclonal); Rabbit anti‐adenomatous polyposis coli (APC) Ab (Cat#: AF0547; Affinity Biosciences); Rabbit anti‐Phospho Catenin Beta (Ser33/37/Thr41) Ab (Cat#: DF2989; Affinity Biosciences); Rabbit anti‐Phospho Akt (Thr308) Ab (Cat#: 13038; Cell Signaling Technology); Rabbit anti‐Phospho Akt (Ser473) Ab (Cat#: A5030; Bimake.Com); Mouse anti‐β‐actin Ab (Cat#: A5316‐100); Mouse anti‐β‐tubulin Ab (Cat#: sc‐5274); Mouse anti‐GAPDH Ab (Cat#: 30201ES60; YEASEN); Rabbit anti‐Beta‐Catenin Ab (Cat#: A5038; Bimake.Com); Mouse anti‐Strep II Tag Ab (Cat#: YFMA0054; YI FEI XUE BIOTECHNOLOGY); Mouse anti‐Flag Tag Ab (Cat#: YFMA0036; YI FEI XUE BIOTECHNOLOGY).

The following secondary Abs were used: TRITC‐conjugated Donkey anti‐Mouse IgG (Code: 715‐025‐150) and Cy2‐conjugated Donkey anti‐Rabbit IgG (Code: 711‐225‐152) were purchased from Jackson ImmunoResearch Laboratory. Horseradish peroxidase (HRP)‐conjugated goat anti‐rabbit IgG and HRP‐conjugated goat anti‐mouse IgG were purchased from Vazyme. Donkey Anti‐rabbit IgG/Gold (35 nm, Cat#: bs‐0295D‐Gold) and Donkey Anti‐rabbit IgG/Gold (15 nm, Cat#: bs‐0295D‐Gold) were purchased from Bioss.

### 2.3 Oocytes collection and culture

Fully grown immature oocytes arrested in GV stage were obtained from the ovaries of 3‐4‐week‐old ICR female mice. Oocytes were released from the ovary by puncturing the follicles with a hypodermic needle. Surrounding cumulus cells were removed by repeatedly pipetting. Every 50 isolated denuded oocytes were placed in 100 µL droplets of culture medium under mineral oil in plastic dishes (BD). The culture medium was MEM+ (MEM with 0.01 mmol/L EDTA, 0.23 mmol/L Na‐pyruvate, 0.2 mmol/L penicillin/streptomycin, 3 mg/mL bovine serum albumin [BSA]) containing 20% foetal bovine serum (FBS). Oocytes were cultured at 37.0°C, 5% O_2_, 5% CO_2_ in a humidified atmosphere.

### 2.4 Antibody transfection

Chariot™ Protein Delivery Reagent (Active Motif) was used for antibody transfection. Abs for transfection have been thoroughly buffer exchanged (>10^4^ dilutions of original buffer) into PBS/50% glycerol with a size‐exclusion spin column (cutoff, 100 kD_a_; spin speed, 5000 rpm; Millipore, Sigma) to remove antiseptics (usually N_a_N_3_) in the original package. In brief, two tubes, one of which contained 1 µL chariot (1 mg/mL in 50% DMSO) in 5 µL sterile water, the other 1 µg Ab in PBS (final volume, 6 mL), were first set up. Solutions from the two tubes were mixed gently and incubated at room temperature for 30 minutes to allow for the formation of the chariot‐IgG complex. The complex solution was added into a 100 mL MEM + drop that contained 50 oocytes. After 12‐14 hours of treatment, oocytes were washed to remove the complex‐containing MEM+. Oocytes were then prepared for WB or transferred into milrinone‐free MEM+ and cultured for 8‐16 hours for phenotype analysis‐related experiments below. During the entire treatment, 2.5 mmol/L milrinone was included to prevent the resumption of meiosis.

### 2.5 Small interfering RNA knockdown

Sequences of all DNA templates for small interfering RNA (siRNA) were designed by using the online siRNA design service, BLOCK‐iT RNAi Designer (http://rnaidesigner.invitrogen.com/rnaiexpress/) with some modification. Sequence specificity was confirmed by a blast homology search. The control template is a mock sequence that was not complementary to any mouse mRNA. Detailed sequence information is in Tables S1 and S2.

Small interfering RNAs were produced by using the T7 RiboMAX Express RNAi System (Promega) according to the manufacturer's instructions. In brief, for each double‐stranded siRNA against one of the four target gene regions, two pairs of synthesized complementary single‐stranded DNA oligonucleotides were first annealed to form 2 double‐stranded DNA templates. Subsequently, two complementary single‐stranded siRNAs were separately synthesized in accordance with these two templates and then annealed to constitute a final double‐stranded siRNA. siRNA was then purified by conventional phenol/chloroform/isopropanol precipitation, which was aliquoted and stored at −80°C after a quality check on an agarose gel. A ready‐to‐use siRNA mixture was set by mixing siRNAs against four distinct target regions at an equal molar ratio to a final concentration of 5 µmol/L.

For siRNA transfection, we used the N‐TER Nanoparticle siRNA Transfection System (Millipore, Sigma). In brief, two tubes, one of which contained 1.1 µL N‐TER^TM^ nanoparticles in 5.15 µL nuclease‐free water (Acros Organics), the other 1.625 µL of siRNA (5 µmol/L) mixture in 4.625 µL siRNA dilution buffer (provided by the kit), were set up. These were then gently mixed and incubated at room temperature for 20 minutes. The siRNA‐nanoparticle complex solution was then added into a 100 µL medium drop that contained 50 oocytes. After a 12‐14 hour treatment, oocytes were washed to remove the nanoparticle‐containing medium. After 1‐2 hours, another 1 or 2 rounds of siRNA treatment were performed, depending on how difficult it was to knock down the target significantly. During the entire siRNA treatment, typically 36‐44 hours long, 2.5 mmol/L milrinone was included to prevent the resumption of meiosis. Oocytes were then collected for experiments described in data analysis and statistics.

### 2.6 In vitro fertilization

Spermatozoa were obtained from the epididymis of 10‐18‐week‐old B6‐DBA2 F1 male mice and were then capacitated in 1 mL MEM + for 1 hour. Subsequently, 10 µL of the suspension containing 5‐10 x 10^6^/mL spermatozoa was added to 490 µL MEM + medium, and oocytes washed off FBS were added. Five hours later, the spermatozoa remaining on the surface of oocytes were washed off by pipetting. After another 4 hours, the oocytes were processed for immunoassaying to determine the frequency of successful fertilization, by the identification of the formation of pronuclei.

### 2.7 Immunofluorescence

After washed in PBS with 0.05% polyvinylpyrrolidone (PVP) briefly, oocytes were permeabilized in 0.5% Triton X‐100/PHEM (60 mmol/L PIPES, 25 mmol/L HEPES pH 6.9, 10 mmol/L EGTA, 8 mmol/L MgSO_4_) for 5 minutes and washed three times rapidly in PBS/ PVP. Next, the oocytes were fixed in 3.7% paraformaldehyde (PFA)/PHEM for 20 minutes and blocked in 1% BSA/PHEM with 100 mmol/L glycine at room temperature for 1 hour. Then, the oocytes were incubated at 4°C overnight with primary antibody diluted in blocking buffer. After being washed three times (10 minutes each) in PBS with 0.05% tween‐20, the oocytes were incubated at room temperature for 45 minutes with secondary antibody diluted in blocking buffer (1:750 in all cases). Finally, chromosomes were stained by 10 µg/mL Hoechst 33342 (Sigma) for 10 minutes and the oocytes were mounted onto a slide with mounting medium (0.5% proposal gallate, 0.1 mol/L Tris‐Hcl, pH 7.4, 88% Glycerol) and covered with a cover glass (0.13‐0.17 µm thick). To maintain the dimension of the oocytes, two strips of double‐stick tap (90 µm thick) were stuck between the slide and cover glass. The oocytes were examined with an Andor Revolution spinning disk confocal workstation (Oxford instruments).

### 2.8 Chromosome spread

Oocytes were exposed to Tyrode's buffer (pH 2.5) for about 40 seconds to remove zona pellucida and then fixed in a drop of 1% PFA with 0.15% Triton X‐100 (pH = 9.2) on a glass slide. Kinetochore and chromosome were stained as that of immunofluorescence. The Andor Revolution spinning disk confocal workstation was used to examine chromosome and kinetochore numbers in oocytes.

### 2.9 Immunoprecipitation

Five micrograms Rabbit anti‐Fam70a antibody, Rabbit anti‐Wnt5a, Rabbit anti‐beta‐catenin or Rabbit IgG was first coupled to 30 μL protein A/G beads (Macgene) for 4 hours at 4°C on a rotating wheel in 250 μL immunoprecipitate (IP) buffer (20 mmol/L Tris‐HCl, pH 8.0, 10 mmol/L EDTA, 1 mmol/L EGTA, 150 mmol/L NaCl, 0.05% Triton X‐100, 0.05% Nonidet P‐40, 1 mmol/L phenylmethylsulfonyl fluoride) with 1:100 protease inhibitor (Sigma) and 1:500 phosphatase inhibitor (Sigma). Meanwhile, 600 oocytes were lysed and ultrasonicated in 250 µL IP buffer and then pre‐cleaned with 30 µL protein A/G beads for 4 hours at 4°C. After that, protein A/G‐coupled Rabbit IgG or specific antibody was incubated overnight at 4°C with pre‐cleaned oocyte lysate supernatant. Finally, after being washed three times (10 minutes each with 250 µL IP buffer), the resulting beads with bound immunocomplexes were subjected to SDS‐PAGE and silver staining.

### 2.10 NIH3T3 cell culture and DNA transfection

NIH3T3 cells were cultured in Dulbecco's modified Eagle's medium (Gibco) with 5% FBS and 1% antibiotics in 5% CO_2_ humidified atmosphere at 37°C. The Flag‐Fam70A and Strep II‐Wnt5a were cloned into the pcDNA3.1(+) vector independently. For transfection experiments, NIH3T3 cells were seeded into 6‐well plates 24 hours prior to transfection at a density of 60%‐70% confluence. For each well, cells were co‐transfected with 3 ug of pcDNA3.1(+)‐Flag‐Fam70A and 3 μg pcDNA3.1(+)‐Strep II‐Wnt5a using Lipofectamine 2000 transfection reagent (Fisher Scientific) and Opti‐MEM (Fisher Scientific) according to the manufacturer's instruction. Forty eight hours later, cells were collected for immunoprecipitation.

### 2.11 Silver staining and characterization of Fam70A‐interacting proteins

For silver staining, immunocomplex beads from the control IgG or Fam70A antibody group were boiled in protein sample buffer, and supernatants were separated side by side on an SDS‐PAGE gel. The gel was firstly fixed overnight in 10% acetic acid and 40% ethanol and sensitized for 30 minutes at room temperature with a fresh sensitizing solution (30% ethanol, 0.2% Na_2_S_2_O_3_, 0.314% Na_2_S_2_O_3_∙5H_2_O and 6.8% sodium acetate). Following being washed three times with water for 5 minutes each, the gel was then stained for 20 minutes at room temperature in staining solution (0.25% AgNO_3_, 0.02% of fresh 37% formaldehyde solution), washed with water for 2.5 minutes and developed for about 5‐10 minutes in developing solution (2.5% NaCO_3_, 0.02% of fresh 37% formaldehyde solution). Finally, 0.4% glycine was used to stop the developing reaction.

To identify Fam70A‐interacting proteins, silver‐stained control or Fam70A was compared carefully, and those bands with the remarkably higher grey level in the Fam70A lane were cut out one by one and stored in protease‐free tubes with 10% ethanol. The selected bands, which were potentially Fam70A integrators, were then sent to the Testing and Analysis Center (Nanjing Medical University) to undergo matrix‐assisted laser desorption/ionization time of flight mass spectrometry (MALDI‐TOF MS, simplified as MALDI). The identity of each protein was given in peptide mass fingerprinting searches in Mascot (http://www.matrixscience.com/mascot/cgi/search_form.pl?FORMVER=2&SEARCH=PMF).

### 2.12 Yeast two‐hybrid assays

The coding sequence of Fam70A was cloned into pGADT7, and the coding sequences of Wnt5a were cloned into pGBKT7. Two plasmids were together transformed into yeast strain AH109. Empty vectors were used as negative controls. The transformed yeast was grown on synthetically defined SD/‐Trp‐Leu dropout medium plates for 2‐4 days. The yeast cells with co‐transformation of the two fusion plasmids were further dropped on SD/‐Trp‐Leu‐His‐Ade dropout medium plates for further verification of the interactions.

### 2.13 Immunogold‐electron microscopy of oocytes

Metaphase I oocytes were blocked with 1% BSA, incubated with Fam70A Ab (4°C, overnight) and next labelled with Donkey anti‐rabbit IgG/Gold (35 nm, room temperature for 2 hours). Then, oocytes were re‐blocked with 1% BSA (to eliminate the cross‐reaction between two antibodies), incubated with Wnt5a Ab (4°C, overnight) and then labelled with Donkey anti‐rabbit IgG/Gold (15 nm, room temperature for 2 hours).

After being labelled, oocytes were fixed in 2.5% glutaraldehyde for 2 hours at 4°C, washed three times with PBS and stained with eosin for 2 minutes to facilitate the oocyte positioning by eye. Oocytes were then placed in 2% agarose and spun for 5 minutes at 13 000 rpm and held overnight at 4°C. The next day, the agarose piece with eosin‐stained oocytes (red in the piece) was trimmed and sent to the Testing and Analysis Center (Nanjing Medical University) for sample preparation for transmission electron microscopy. Electron microscopy pictures were obtained with a transmission electron microscope (FEI Tecnai G2 Spirit Bio Twin; Thermo Fisher Scientific).

### 2.14 Mitochondrial staining, Lysosome staining and ATP measurements

For mitochondrial staining, oocytes were incubated in HEPES containing 100 nmol/L MitoTracker (Cat#: M7521, Invitrogen) and 10 μg/mL Hoechst 33342 (Sigma) for 30 minutes. Images were taken with an Andor Revolution spin disk confocal workstation.

For lysosome staining, the working concentration of LysoTracker (Cat#: C1046, Beyotime) was 50 nmol/L (diluted by HEPES). Oocytes were incubated in LysoTracker working solution and 10 μg/mL Hoechst 33342 (Sigma) for 30 minutes. Images were taken with an Andor Revolution spin disk confocal workstation.

For ATP measurements, oocytes were first lysed with 100 μL ATP lysis solution (Cat#: S0026; Beyotime) on ice. The samples were then detected by enzyme‐labelled instrument Synergy2 (BioTek) to evaluate ATP level.

### 2.15 Data analysis and statistics

All experiments were repeated at least three times. Measurement of Western blot and confocal images was conducted with Image J. (National Institutes of Health). Data are presented as mean ± SEM. Statistical comparisons between 2 groups were made with the Student's *t* test of the Excel program (Microsoft). Multiple comparisons were made by using the Kruskal‐Wallis one‐way nonparametric ANOVA (Prism; GraphPad Software). Values of *P* < .05 were considered statistically significant.

## 3 RESULTS

### 3.1 The "female fertility factor" Fam70A is important for the meiotic progression of IVM mouse oocytes

The integral membrane protein Fam70A was previously identified as a "female fertility factor" and is the only Fam70 family member with a predominant expression in the ovaries.^7^ Therefore, we hypothesized that this protein may be important for oocyte meiosis.

**FIGURE 1:**
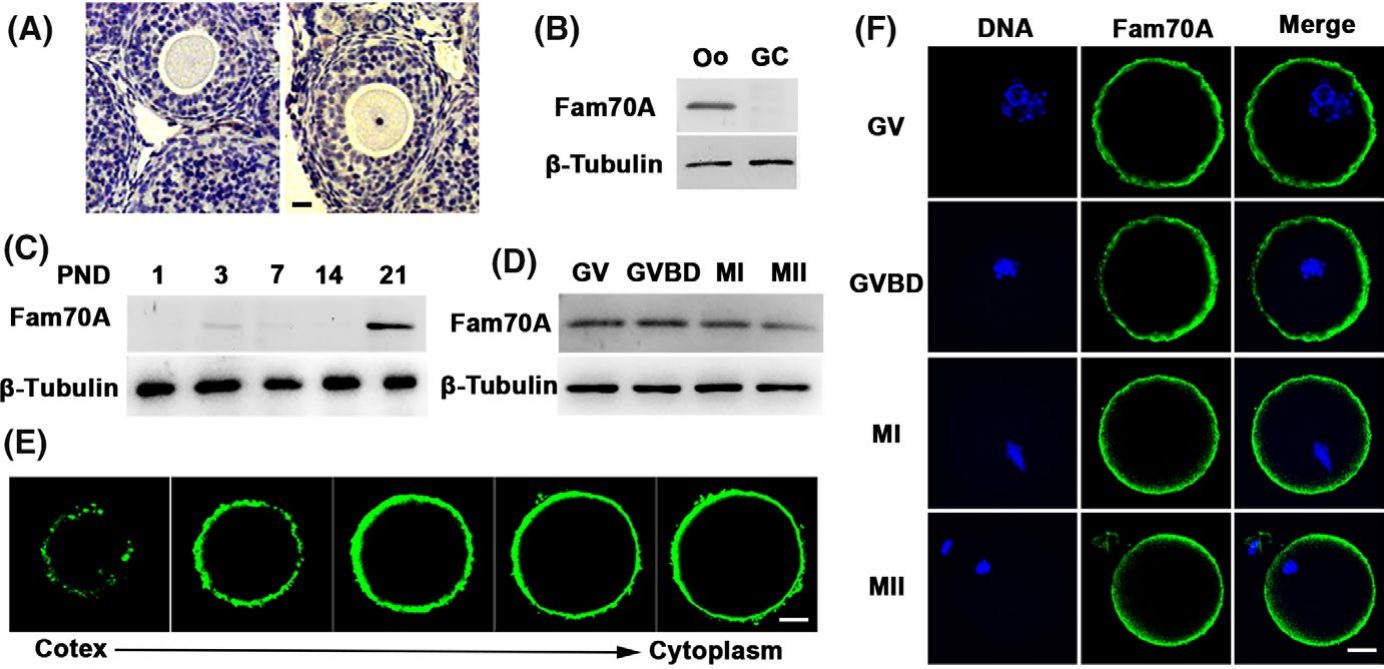
The “female fertility factor” Fam70A is enriched on the oocyte membrane. A, Immunohistochemistry showed that Fam70A was enriched on the oocyte membrane of growing oocytes. Fam70A was developed in brown; DNA was stained with haematoxylin. Two secondary follicles were shown. B, Western blot showed that Fam70A was more abundant in oocytes (Oos) than in granular cells (GCs). C, Western blot showed that the Fam70A protein level remarkably elevated as follicles were initially recruited (PND 21). D, Western blot showed that the Fam70A protein level kept constant during meiosis. E, Z‐slices of Fam70A immunofluorescent image showed that Fam70A was enriched on the oocyte membrane. F, Immunofluorescence showed that Fam70A remained on the oocyte membrane during meiosis. Scale bar, 20 µm. GV, germinal vesicle; GVBD, germinal vesicle breakdown; MI, metaphase I; MII, metaphase II; PND, post‐natal day

We first examined the localization and expression of Fam70A within mouse oocytes. Results showed that Fam70A was more highly expressed within oocytes than granulosa cells (Figure 1A,B). The expression in the ovaries increased sharply as follicles were initially recruited (PND 21) (Figure 1C). During meiosis, Fam70A exhibited a constant expression level (Figure 1D) and was exclusively concentrated on oocyte membranes (Figure 1E,F).

**FIGURE 2:**
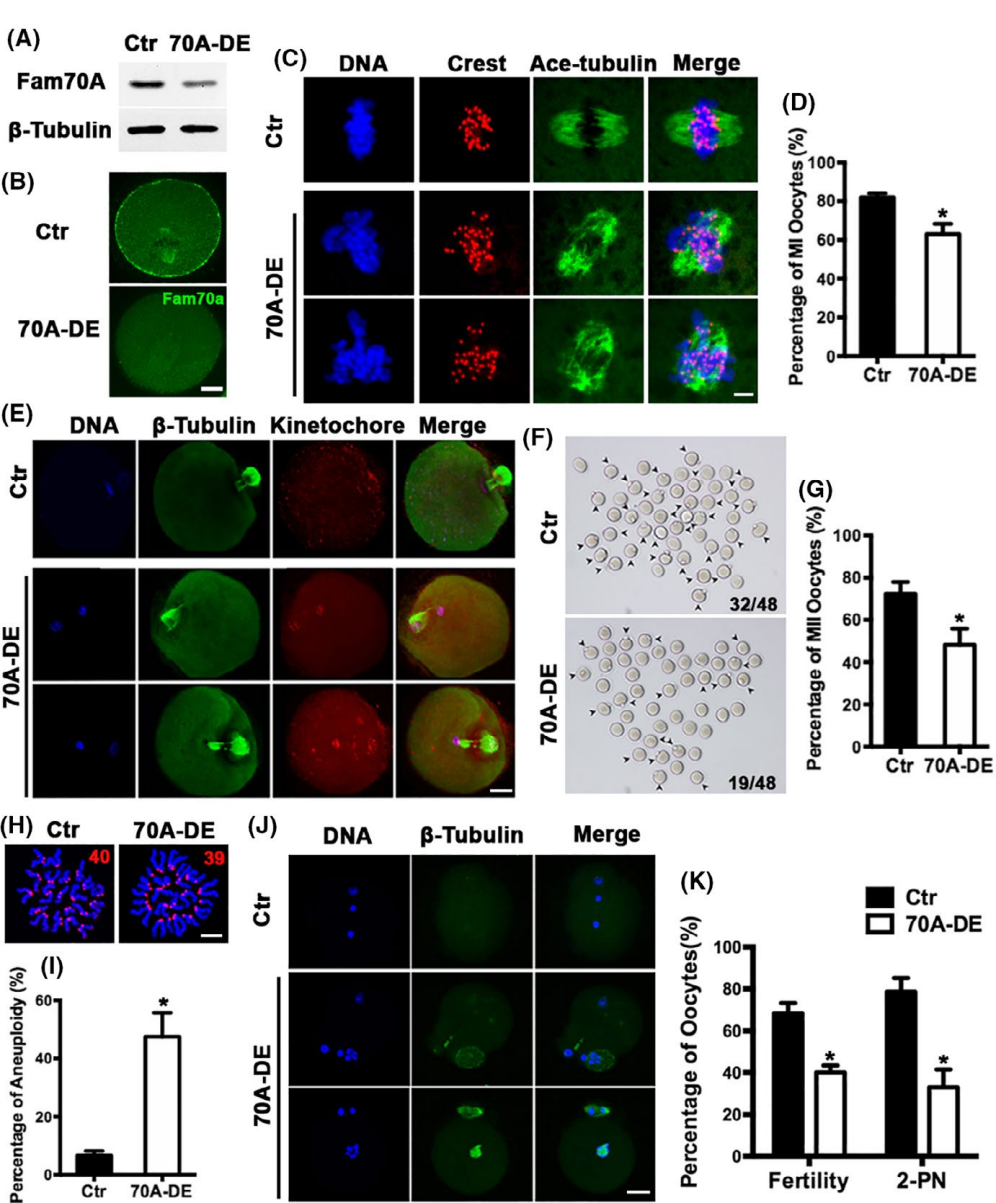
Fam70A is important for normal meiosis and fertilization of in vitro maturation (IVM) mouse oocytes. A, Western blot showed that Fam70A could be remarkably reduced by chariot‐mediated antibody transfection. B, Immunofluorescence showed that Fam70A staining was remarkably reduced by chariot‐mediated antibody transfection. C, Immunofluorescence showed that Fam70A depletion (70A‐DE) remarkably disrupted chromosome congression at 7.5 h of IVM. D, Quantification of (C). E, Immunofluorescence showed that Fam70A depletion retarded 1 pb (first polar body) extrusion at 12 h of IVM. F, Representative bright‐field images of oocytes at 14.5 h of IVM. Numbers in the images indicate the number of MII oocytes/number of total oocytes. G, Quantification of (F). H, Immunofluorescence showed that Fam70A depletion remarkably increased oocytes with aneuploidy after Fam70A depletion. I, Quantification of (H). J, Immunofluorescence of in vitro fertilization oocytes showed that Fam70A depletion remarkably reduced fertility rate and 2‐PN (pro‐nucleus) rate. K, Quantification of (J). Except for (B) (Fam70A in green), tubulin in green, kinetochores in red, DNA in blue. Scale bar in (C) and (H), 5 µm; Scale bar in other panels, 20 µm. *P < .05. MII, metaphase II

Next, we examined whether Fam70A knockdown affected oocyte meiosis. Fam70A protein levels were remarkably reduced with peptide‐mediated antibody transfection (Figure 2A,B), as previously described.^14^, ^15^ We found that at 7.5 hours of in vitro maturation (IVM), spindle organization was severely disrupted and chromosome congression was impeded. Furthermore, the percentage of MI oocytes was remarkably decreased (Figure 2C,D, percentage of MI oocytes, Ctr vs Fam70A‐DE, 82% vs 63%). At 12 hours of IVM, polar body extrusion was severely impeded (Figure 2E). At 14.5 hours of IVM, the maturation rate (first polar body extrusion) remarkably decreased (Figure 2F,G; percentage of MII oocytes, Ctr vs Fam70A‐DE, 72.37% vs 48.21%). Moreover, the percentage of MII oocytes with aneuploidy dramatically increased, potentially due to the disrupted spindle organization and aberrant chromosome alignment (Figure 2H,I, percentage of MII oocytes with aneuploidy, Ctr vs Fam70A‐DE, 6.7% vs 47.5%). Finally, in vitro fertilization results showed that Fam70A depletion dramatically reduced the fertilization rate (Figure 2J,K, percentage of fertilized oocytes, Ctr vs Fam70A‐DE, 68.32% vs 40.09%) and 2‐pronucleus (PN) rate (Figure 2J,K, percentage of fertilized oocytes with 2‐PN, Ctr vs Fam70A‐DE, 78.70% vs 33.02%).

These results indicated that Fam70A, an integral membrane protein that was screened as a "female fertility factor," was important for the meiotic progression of IVM oocytes.

### 3.2 Fam70A is important for oocyte quality

**FIGURE 3:**
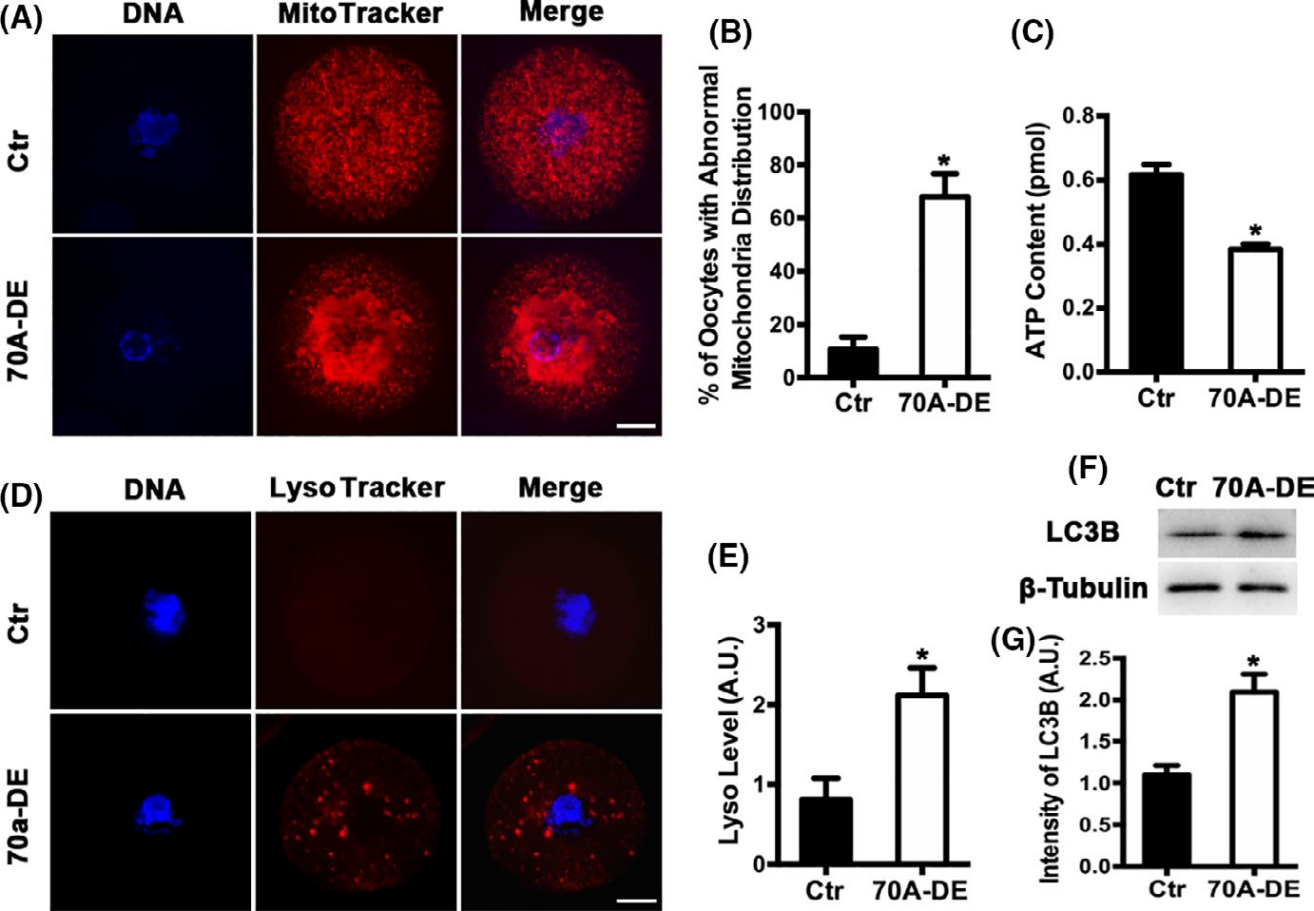
Fam70A is important for oocyte quality. A, Fluorescent live tractor staining showed that Fam70A depletion caused mitochondria over‐aggregation. Mitochondria in red, DNA in blue. B, Quantification of (A). C, Fam70A depletion remarkably reduced ATP concentration within oocytes. D, Fluorescent live tractor staining showed that Fam70A depletion remarkably increased lysosome aggregation. Lysosome in red, DNA in blue. E, Quantification of (D). F, Western blot showed that LC3B remarkably increased in Fam70A‐depleted oocytes. G, Quantification of (F). Scale bar, 20 µm. *P < .05

The discrepancy between MII, fertilization and 2‐PN rates suggested that certain Fam70A‐depleted MII oocytes were not fully competent. Mitochondria are responsible for ATP production, and mitochondrial dysfunction may decrease oocyte viability.^16^ Autophagy is an important process wherein LC3B‐labelled cellular waste is encapsulated in autosomes and subsequently transported to the lysosomes for degradation. Decreased autophagy prevents the removal of waste, thereby reducing oocyte viability.^17^ Therefore, mitochondrial dysfunction and autophagy were further investigated. First, mitochondria distribution analysis showed that mitochondria formed several large aggregates in Fam70A‐depleted oocytes (Figure 3A,B). Furthermore, the ATP level was remarkably reduced (Figure 3C; Ctr vs Fam70A‐DE, ATP conc., 0.62 vs 0.38). Second, Fam70A depletion caused the abnormal aggregation of lysosomes (Figure 3D,E), and the LC3b level increased in Fam70A‐depleted oocytes (Figure 3F,G).

Over‐aggregation of mitochondria or lysosomes suggested that mitochondrial function and normal autophagy were severely disrupted in Fam70A‐depleted oocytes, as also demonstrated by decreased ATP and increased LC3B levels, and may adversely affect oocyte quality.

### 3.3 Fam70A directly interacts with oocyte‐predominant Wnt5a

**FIGURE 4:**
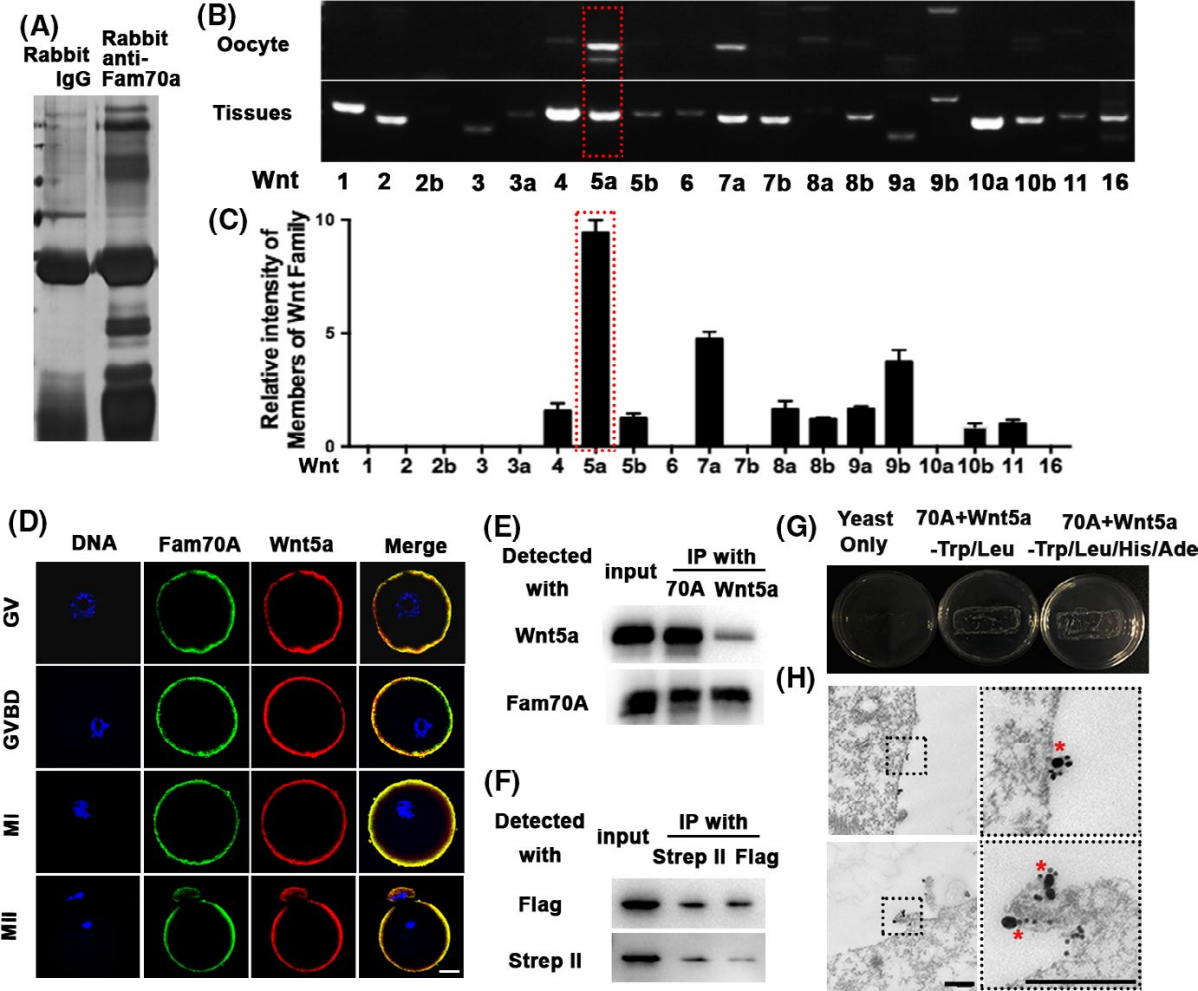
Fam70A interacts directly with oocyte‐predominant Wnt5a. A, SDS‐PAGE and silver staining of Fam70A antibody or control IgG IP complex showed many distinct bands; we cut and sent these distinct bands for IP‐MALDI to identify the potential interacting proteins of Fam70A (see also Figure S2A). B, Among all Wnt members, we found that Wnt5a (red dot‐line square) is the most abundant within mouse oocytes. C, Quantification of (B). D, Immunofluorescence showed that the Fam70A signal on the oocyte membrane highly overlapped with Wnt5a. Fam70A in green, Wnt5a in red, DNA in blue. E, Co‐IP in oocyte lysate showed that endogenous Fam70A and Wnt5a interacted with each other. F, Co‐IP in the lysate of NIH3T3 cells co‐transfected with Fam70A and Wnt5A plasmids showed that exogenous Fam70A and Wnt5a also interacted with each other. G, Yeast 2‐hybrid assays showed that Fam70A directly binds Wnt5a on both Trp/Leu‐deficient and Trp/Leu/His/Ade‐deficient Agar plate. H, Immuno‐EM showed that Fam70A localized close (<20 nm) to Wnt5a on oocyte membrane or microvillus. Fam70A and Wnt5a were labelled with 35 and 15 nm gold particles, respectively. Scale bar in E, 20 µm. Scale bar in F, 400 nm. GV, germinal vesicle; GVBD, germinal vesicle breakdown; MI, metaphase I; MII, metaphase II

Next, we performed IP‐MALDI to identify proteins that interact with Fam70A (Figure 4A; Figure S2A). A PubMed search revealed that four proteins, which were specifically enriched within the Fam70A IP complex but not in the control, might be correlated with the Wnt‐β‐catenin pathway (Figure S2B; Table S1). This suggested that Fam70A may interact with Wnt signalling. By systematic comparison of the abundance of all Wnt family members, we found that Wnt5a is the most abundant member within mouse oocytes (Figure 4B,C). Co‐staining showed that Fam70A and Wnt5a co‐localized (Figure 4D). Co‐IP in oocytes and transfected NIH3T3 cells showed that the two proteins interacted with each other (Figure 4E,F), while a yeast two‐hybrid assay confirmed their direct interaction (Figure 4G). Furthermore, immunogold‐electron microscopy showed that Fam70A and Wnt5a were located to each other (<20 nm) on oocyte membranes and microvilli (Figure 4H). These results indicated that Fam70A may directly bind Wnt5a during meiosis.

### 3.4 Fam70A and Wnt5a regulate the APC level and microtubule stability

**FIGURE 5:**
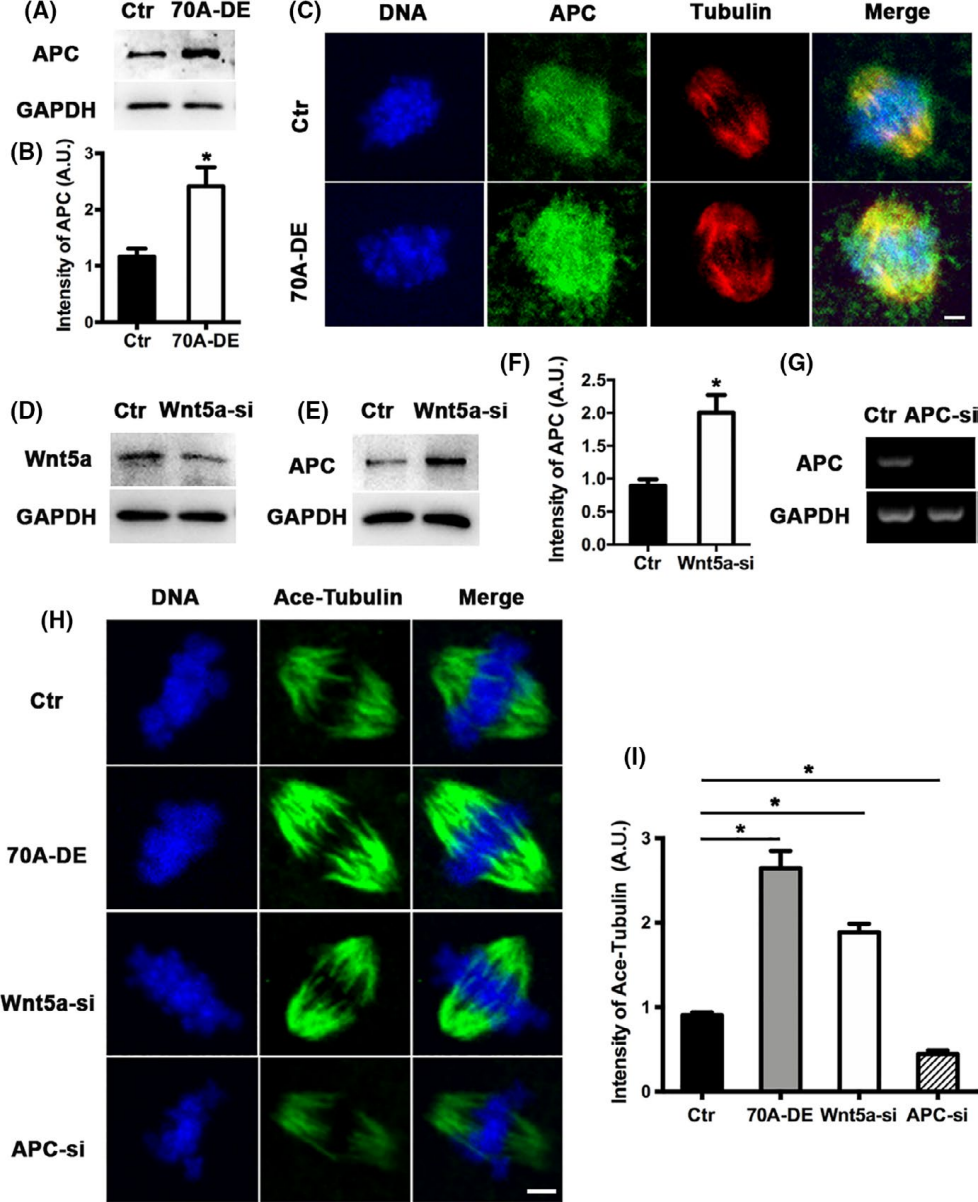
Fam70A and Wnt5a regulate adenomatous polyposis coli (APC) level and MT stability. A, Western blot showed that APC protein remarkably increased after Fam70A depletion. B, Quantification of (A). C, Immunofluorescence showed that APC was enriched within spindles and remarkably increased after Fam70A depletion. APC in green, tubulin in red, DNA in blue. D, Western blot showed that Wnt5a was efficiently reduced by siRNA (Table S2). E, Western blot showed that APC remarkably increased after Wnt5a knockdown. F, Quantification of (E). G, RT‐PCR showed that APC was efficiently reduced by siRNA (Table S3). H, Fam70A depletion and Wnt5a knockdown both remarkably increased acetylated tubulin level within spindles, while APC knockdown remarkably reduced acetylated tubulin level. Acetylated tubulin in green, DNA in blue. I, Quantification of (H). Scale bar, 5 µm. *P < .05

The APC protein is an important intermediate component of the canonical Wnt signalling pathway. APC regulates the dynamics of microtubules and microfilaments,^18^, ^19^, ^20^ making it an ideal candidate for modulating meiotic spindle formation and meiosis. Depletion of either Fam70A or Wnt5a remarkably increased the APC protein level (Figure 5A‐F), and correspondingly, acetylated tubulin levels (Figure 5H,I). By contrast, APC knockdown remarkably decreased the level of acetylated tubulin (Figure 5G‐I). These results suggested that Fam70A/Wnt5a may regulate microtubule stability through APC.

### 3.5 Fam70A and Wnt5a regulate β‐catenin activity

**FIGURE 6:**
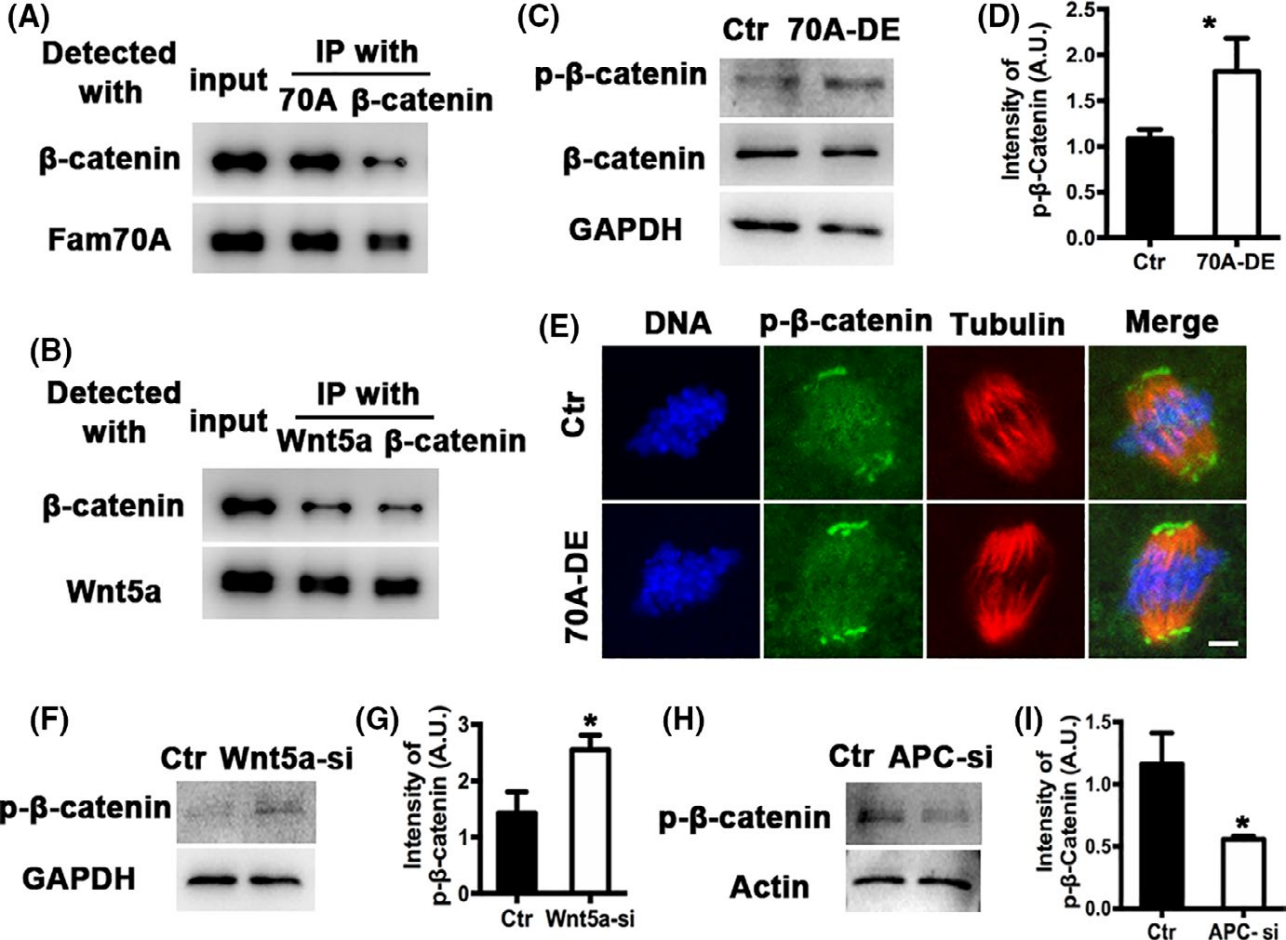
Fam70A and Wnt5a regulate β‐catenin activity. A, Co‐IP showed that Fam70A interacted with β‐catenin within oocytes. B, Co‐IP showed that Wnt5a interacted with β‐catenin within oocytes. C, Western blot showed that Fam70A depletion remarkably increased p‐β‐catenin level. D, Quantification of (C). E, Immunofluorescence showed that Fam70A depletion remarkably increased p‐β‐catenin at spindle poles. F, Western blot showed that Wnt5a knockdown remarkably increased p‐β‐catenin level. G, Quantification of (F). H, Western blot showed that APC knockdown remarkably decreased p‐β‐catenin level. I, Quantification of (H). Scale bar, 5 µm. *P < .05

The transcription factor β‐catenin is a major downstream effector of the canonical Wnt signalling pathway. Therefore, we examined whether Fam70A and Wnt5a regulate its activity. The results showed that either Fam70A or Wnt5a could co‐immunoprecipitate with β‐catenin (Figure 6A,B). Furthermore, depletion of either Fam70A (Figure 6C‐E) or Wnt5a (Figure 6F,G) remarkably increased p‐β‐catenin levels. Conversely, APC knockdown remarkably reduced p‐β‐catenin levels (Figure 6H,I). These results suggested that Fam70A and Wnt5a function in a canonical Wnt‐β‐catenin signalling pathway.

### 3.6 Fam70A and Wnt5a regulate the activity of Akt involved in normal meiosis

**FIGURE 7:**
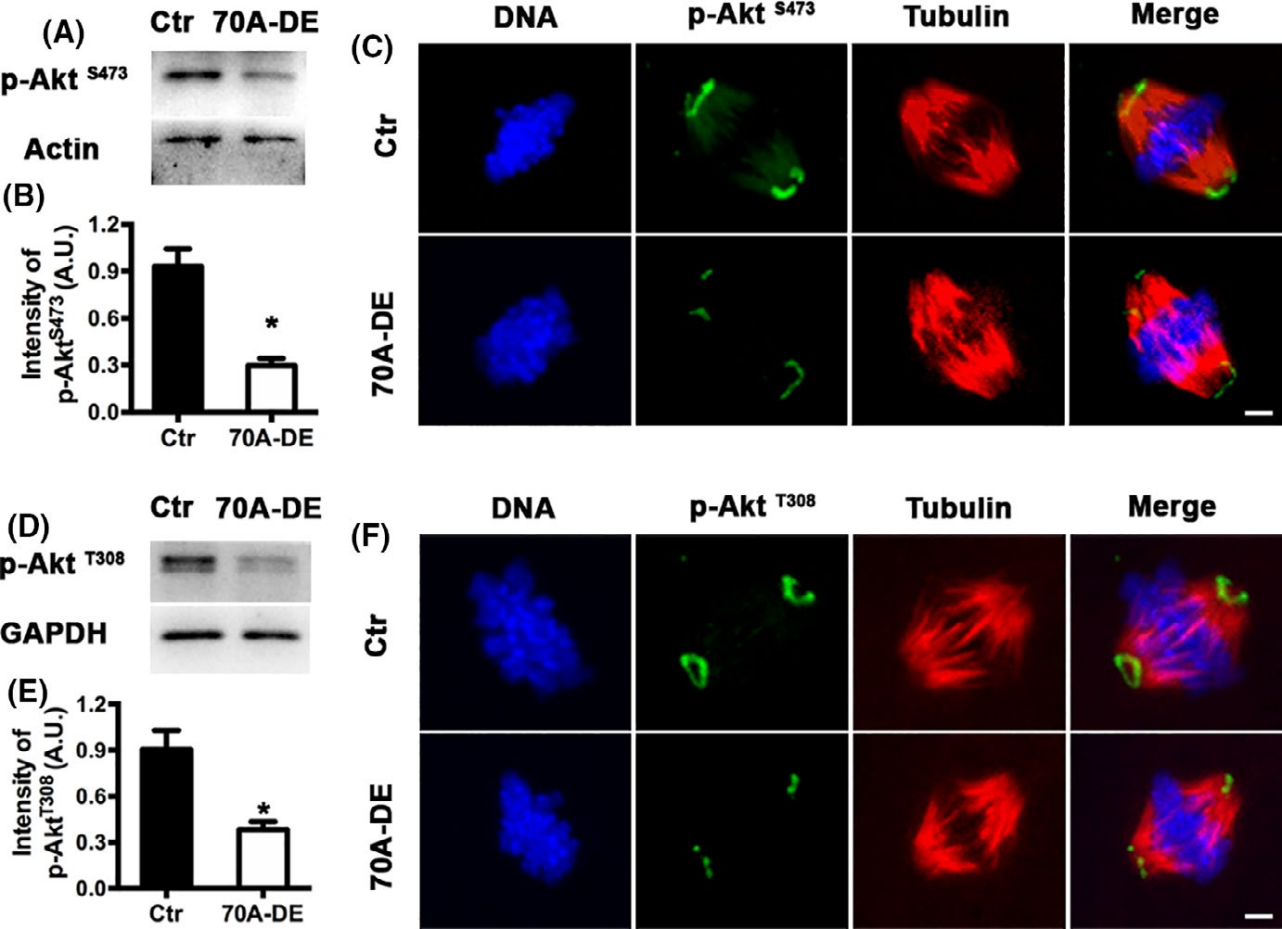
Fam70A regulates Akt activity. A, Western blot showed that Fam70A depletion dramatically decreased p‐Akt^S473^ level. B, Quantification of (A). C, Immunofluorescence showed that Fam70A depletion dramatically decreased p‐Akt^S473^ level within spindles. p‐Akt^S473^ in green, tubulin in red, DNA in blue. D, Western blot showed that Fam70A depletion dramatically decreased p‐Akt^T308^ level. E, Quantification of (D). F, Immunofluorescence showed that Fam70A depletion dramatically decreased p‐Akt^T308^ level within spindles. p‐Akt^T308^ in green, tubulin in red, DNA in blue. Scale bar, 5 µm. *P < .05

In mitotic somatic cells, Wnt regulates several kinases independently of the APC‐β‐catenin pathway, which is termed the “non‐canonical Wnt pathway.”^21^, ^22^ The present study examined whether Fam70A functions through a similar non‐canonical Wnt pathway. Akt is the upstream regulator of several kinases, including mTOR, which regulates follicle activation and oocyte quality.^23^, ^24^ Furthermore, Akt is known to regulate oocyte quality and meiosis.^11^, ^12^, ^13^ Therefore, Akt was investigated in the present study. Western blot and immunofluorescence staining showed that depletion of Fam70A dramatically reduced the phosphorylation (activation) of Akt at both S473 (Figure 7A‐C) and T308 (Figure 7D‐F). These results suggested that Fam70A may also function through a non‐canonical Wnt pathway.

## 4 DISCUSSION

The present study revealed that the "female fertility factor" Fam70A may regulate meiosis through multiple pathways. Furthermore, to the best of our knowledge, we were the first to show that Wnt5a may be the main Wnt family member that regulates the APC expression level and β‐catenin activity during meiosis in oocytes. Moreover, we showed that Fam70A directly binds Wnt5a to regulate both canonical and non‐canonical Wnt signalling pathways.

The roles of integral membrane proteins in meiosis in IVM oocytes have not been widely investigated. One reason may be that it is thought that IVM oocytes do not require membrane‐initiating signals, since IVM cumulus‐free oocytes are able to undergo meiosis in protein‐free media. However, other studies suggested that membrane‐initiating signals are important for in vitro female meiosis. For example, adenosine present in mouse follicular fluid has been shown to prevent oocyte maturation in vitro. However, this effect was not due to adenosine transport into oocytes or adenosine‐induced ATP level increment. It was proposed that adenosine exerts a maturation‐inhibitory effect in oocyte membranes.^25^ Calreticulin, a protein best known as an endoplasmic reticulum chaperone, is present on the extracellular surface of the plasma membrane of mouse oocytes. Calreticulin inhibition altered cortical actin distribution and meiosis resumption independently of intercellular calcium, suggesting that calreticulin mediates transmembrane signalling linked to meiosis resumption.^26^ In the present study, we showed that Fam70A regulated female meiosis by directly binding Wnt5a and initiating downstream signalling. These results, on the one hand, further supported that membrane proteins are important for in vitro female meiosis, and on the other hand, indicated that Wnt5a requires additional membrane proteins to exert its function.

The specific effects that Fam70A exerts on Wnt remain unclear. There are diverse functional subunits called lipid rafts in membranes, which are composed of cholesterol, sphingolipids and diverse proteins, and play important roles in cell signal transduction.^27^ Specifically, Ly6 family protein LY6/PLAUR domain containing 6 (Lypd6) preferentially localizes to the raft membrane domain and positively regulates Wnt signalling.^28^ Lypd6 interacts with the Wnt receptor Frizzled8 and the co‐receptor Lrp6 to control Lrp6 activation (phosphorylation) specifically in membrane rafts. Lypd6 knockdown or mislocalization of the Lypd6 protein to non‐raft membrane domains alters Lrp6 phosphorylation and inhibits Wnt signalling.^28^ Fam70A may serve as a raft protein that assists Wnt activation.

The Wnt‐β‐catenin signalling pathway is highly conserved and serves important roles in early embryonic development, organ formation, tissue regeneration and other physiological processes.^8^, ^9^, ^10^ When Wnt signalling is inactivated, APC complexes phosphorylate β‐catenin, leading to the degradation of β‐catenin, which prevents the nuclear deposition of β‐catenin and related transcriptional regulation.^8^, ^9^, ^10^ The Wnt family includes 16 members and has distinct expression within mouse tissues,^29^ and not surprisingly, different tissues express distinct Wnt members. For example, Wnt9b is predominantly found in the kidney and is important for the development of mesonephric and metanephric tubules, and for caudal extension of the Müllerian duct through the non‐canonical Rho/Jnk branch of the Wnt pathway.^30^ Wnt5a is enriched in lungs, and its expression is significantly higher in non‐small‐cell lung cancer and was positively correlated with that of β‐catenin, vascular endothelial growth factor A and Ki‐67.^31^ Reduction in both canonical and non‐canonical Wnt5a signalling is linked to impaired lung repair in chronic obstructive pulmonary disease.^32^ However, to the best of our knowledge, the specific Wnt member present in oocytes had not been previously determined. In the present study, we found that Wnt5a was the most abundant Wnt protein in oocytes and that Wnt5a knockdown altered the APC level and β‐catenin phosphorylation. Collectively, the results suggested that Wnt5a is the primary Wnt protein involved in oocyte meiosis.

The basic mechanism of the Wnt‐β‐catenin pathway is that β‐catenin acts as a transcription factor that enters the nucleus and modulates transcription. However, in fully grown IVM oocytes, the transcription activity is quiescent, suggesting that β‐catenin is not likely to affect meiosis, but rather serves important roles in subsequent early embryo development.^8^, ^9^, ^10^ However, APC, the intermediate protein of the Wnt‐β‐catenin pathway, may affect in vitro female meiosis. For example, in PTK2 cells, APC was found to bind to microtubules and increased microtubule stability in vivo and in vitro. Deletion of its microtubule binding site from the C‐terminal domain did not eliminate its binding but decreased its capacity to stabilize microtubules.^18^ In MCF‐7 cells, APC is the direct binding partner of ends‐binding 1 and regulates microtubule stability; APC knockdown reduced microtubule stability and cell migration.^19^ The present study revealed that depletion of either Fam70A or Wnt5a remarkably increased APC expression and acetylated tubulin levels within the spindles. APC knockdown remarkably reduced acetylated tubulin levels. Several studies showed that overstable microtubules could impede mitotic cell division.^33^, ^34^ Taken together, the results indicated that APC, as a downstream effector of Fam70A and Wnt5a, may be one of the important modulators of their roles in female meiosis.

In mitotic somatic cells, Wnt functions through not only the canonical Wnt‐β‐catenin pathway, but also the non‐canonical Wnt pathways. The extracellular domain of Ptk7 acts as an important regulator of both non‐canonical Wnt/planar cell polarity (PCP) and canonical Wnt/β‐catenin signalling in zebrafish embryo development.^21^ In basal breast cancer, the archetypal Wnt/PCP protein VANGL PCP protein 2 (VANGL2) is upregulated and is associated with poor prognosis and tumour growth through the VANGL2‐p62/SQSTM1‐JNK pathway.^22^ In the present study, Fam70A depletion also reduced Akt activity. Akt activation is important for mouse oocyte meiosis.^11^, ^12^, ^13^ Thus, Fam70A may also regulate female meiosis through the non‐canonical Wnt pathway.

**FIGURE 8:**
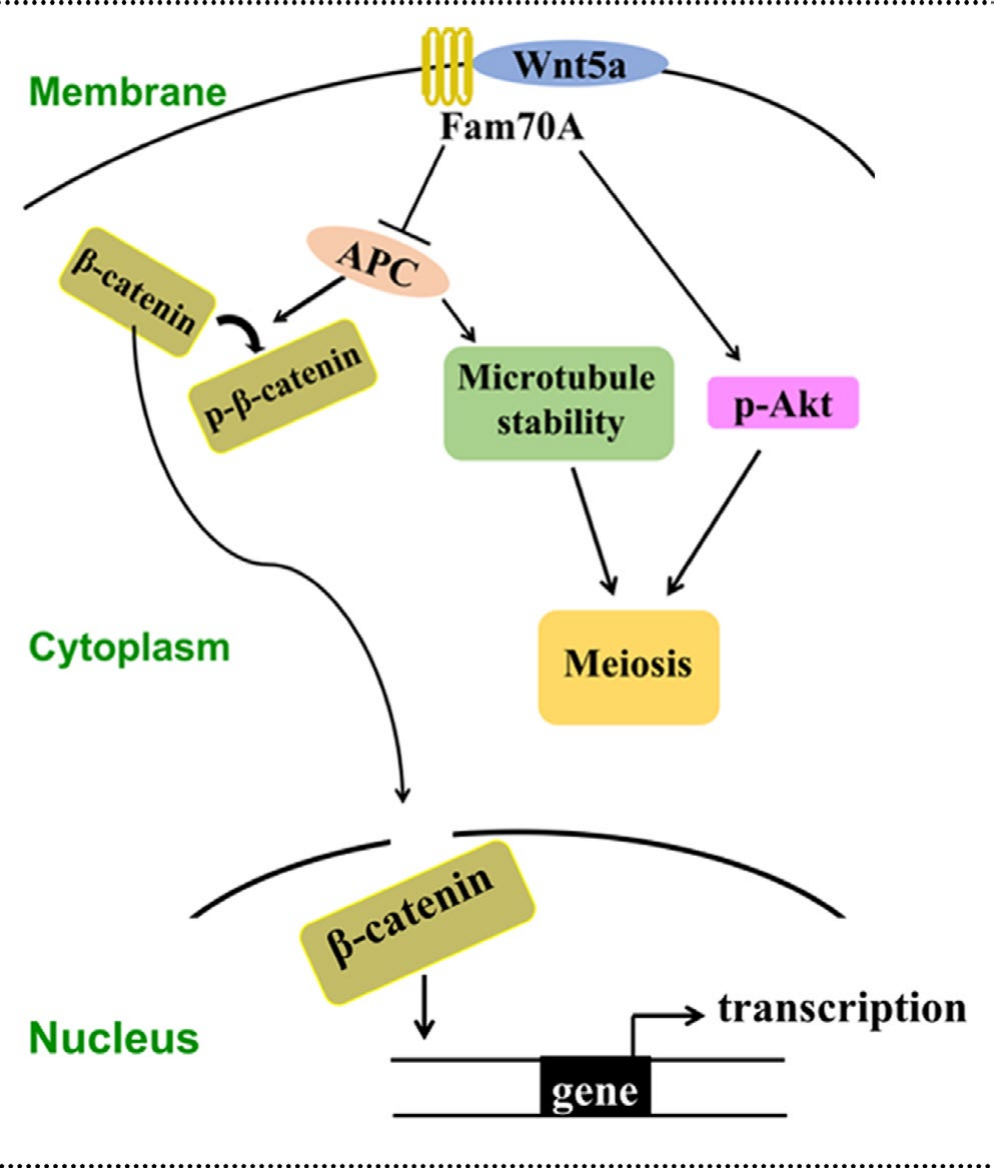
Fam70A working model. At cell membrane, Fam70A directly binds Wnt5a, which results in the inhibition of APC and activation of Akt (p‐Akt). APC promotes the phosphorylation (inactive form) of β‐catenin and stabilizes microtubules as well. β‐Catenin (active form) can get into the nucleus to promote gene transcription (but not in fully grown oocytes). Proper microtubule stability and p‐Akt together promote meiosis and quality of mouse oocytes

In all, the present study revealed that Fam70A is a "female fertility factor" that may bind Wnt5a to regulate the APC level and Akt activity through canonical and non‐canonical signalling, respectively (Figure 8). However, Fam70A may also work independently of Wnt5a to regulate important meiotic kinases, such as Akt. This needs further investigation.

## CONFLICTS OF INTEREST

The authors declare that they have no conflicts of interest.

## AUTHOR CONTRIBUTIONS

Dong Zhang, Chun‐Xiang Zhou, Zhi‐Xia Yang and Jian‐Min Li designed the research; Na‐Na Zhang, Teng Zhang, Wen‐Yi Gao, Xin Wang, Zi‐Bin Wang, Jin‐Yang Cai and Yang Ma performed most of the experiments, data collection and analysis. Cong‐Rong Li, Xi‐Chen Chen, Wen‐Tao Zeng and Fan Hu assisted in these processes. Na‐Na Zhang and Teng Zhang prepared figures under the supervision of Dong Zhang, Chun‐Xiang Zhou, Zhi‐Xia Yang and Jian‐Min Li. Dong Zhang wrote the manuscript with the assistance of Na‐Na Zhang and Teng‐Zhang. Chun‐Xiang Zhou, Zhi‐Xia Yang and Jian‐Min Li proofread and gave advice. All authors read and approved the final manuscript. The authors thank Professor Qing‐Yuan Sun from Institute of Zoology, Chinese Academy of Sciences for valuable advices.

## Supporting information

Fig S1

Fig S2

Table S1

Table S2

Table S3

## Data Availability

The data that support the findings of this study are available from the corresponding author upon reasonable request.
