# Supplementary figures and images for: Fam70A binds Wnt5a to regulate meiosis and quality of mouse oocytes
Source: Cell Prolif. 2020 May 11;53(6):e12825. doi: 10.1111/cpr.12825 (PMC7309945; doi:10.1111/cpr.12825)

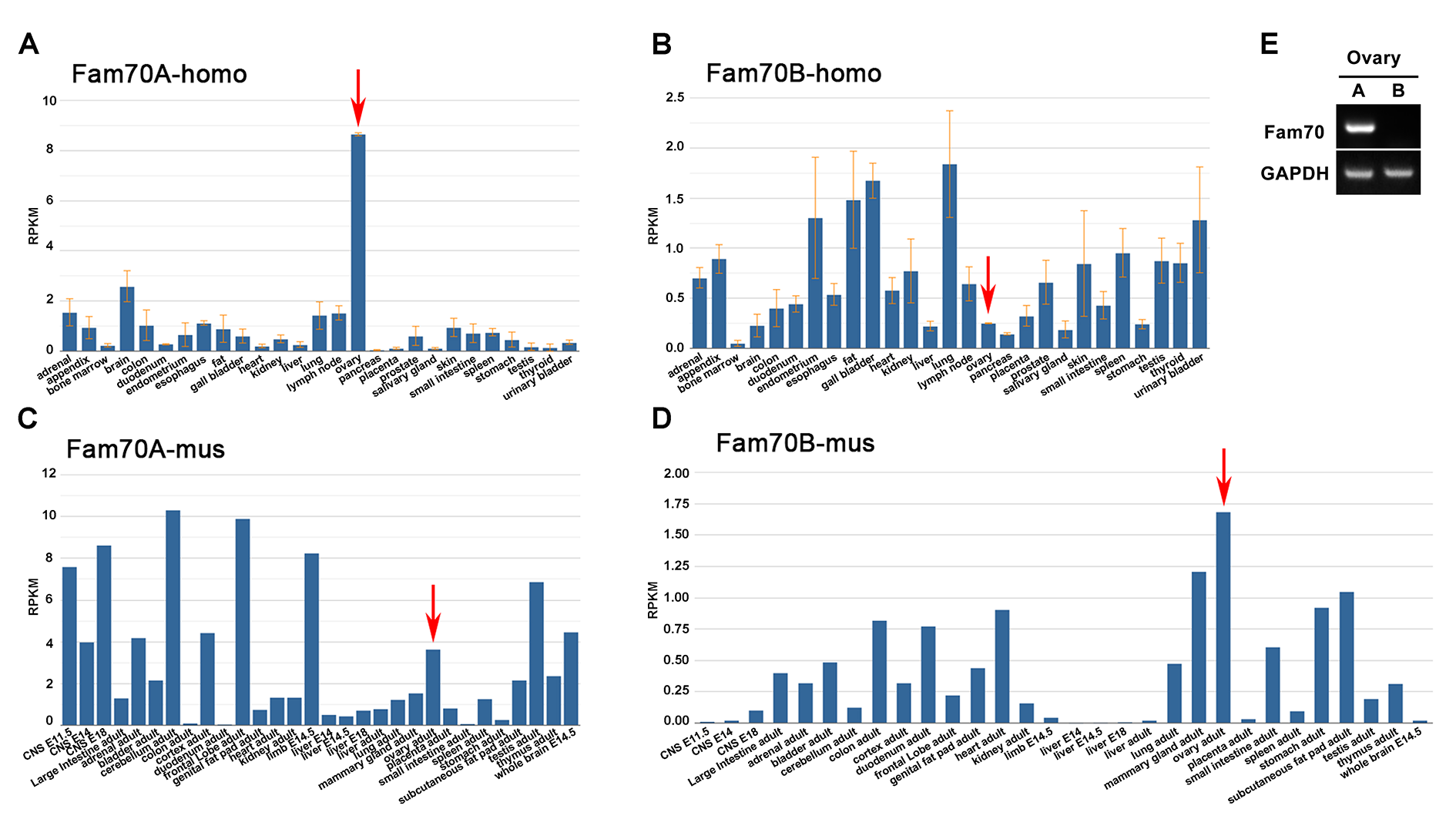

Supplement: Supplementary file 1 — Fig S1 [file CPR-53-e12825-s001.tif]

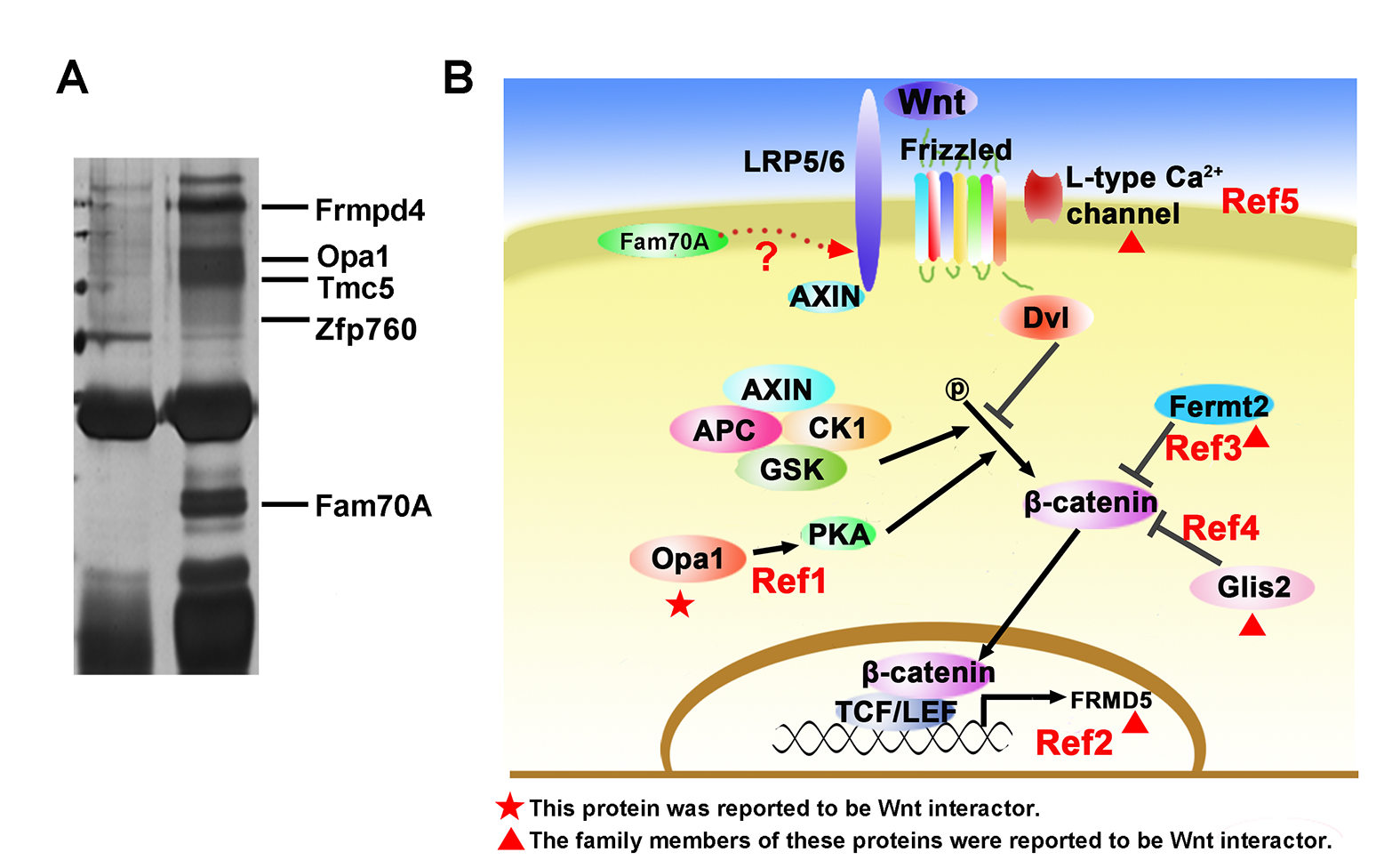

Supplement: Supplementary file 2 — Fig S2 [file CPR-53-e12825-s002.tif]
