# Supplementary material for: Fam70A binds Wnt5a to regulate meiosis and quality of mouse oocytes
Source: Cell Prolif. 2020 May 11;53(6):e12825. doi: 10.1111/cpr.12825 (PMC7309945; doi:10.1111/cpr.12825)
Supplement: Supplementary file 3 — Table S1 [file CPR-53-e12825-s003.doc]

Supplementary table 1. Identified Fam70A interactors and their probable Correlation with Wnt/β-catenin

| **Identified Fam70A interactor** | **Correlation with Wnt/β-catenin** | **Reference** |
| --- | --- | --- |
| Opa1 | Opa1 was reported to interact with Wnt/β-catenin | 1 |
| Frmpd4 | Frmpd4 belongs to the same family with FRMD5 and Fermt2, which were reported to interact with Wnt/β-catenin | 2, 3 |
| Zfp760 | Zfp760 belongs to the same family with Glis2, which was reported to interact with Wnt/β-catenin | 4 |
| Tmc5 | Tmc5 is a transmembrane channel protein like L-type Ca^2+^ channel, which was reported to interact with Wnt | 5 |
