# Supplementary material for: Fam70A binds Wnt5a to regulate meiosis and quality of mouse oocytes
Source: Cell Prolif. 2020 May 11;53(6):e12825. doi: 10.1111/cpr.12825 (PMC7309945; doi:10.1111/cpr.12825)
Supplement: Supplementary file 4 — Table S2 [file CPR-53-e12825-s004.doc]

**Supplementary table 2. DNA oligos for Wnt5a siRNA production.**

| **Target Site** | **DNA templates** |
| --- | --- |
| **683-703^1^** | Oligo1: GGATCCTAATACGACTCACTATAGAAGCAGGCCGTAGGACAGTA ^2^ |
|  | Oligo2: AATACTGTCCTACGGCCTGCTTCTATAGTGAGTCGTATTAGGATCC ^2^ |
|  | Oligo3: GGATCCTAATACGACTCACTATATACTGTCCTACGGCCTGCTTC^2^ |
|  | Oligo4: AAGAAGCAGGCCGTAGGACAGTATATAGTGAGTCGTATTAGGATCC^2^ |
| **803-823^1^** | Oligo1: GGATCCTAATACGACTCACTATAGATGCCCTCAAGGAGAAGTAT ^2^ |
|  | Oligo2: AAATACTTCTCCTTGAGGGCATCTATAGTGAGTCGTATTAGGATCC^2^ |
|  | Oligo3: GGATCCTAATACGACTCACTATAATACTTCTCCTTGAGGGCATC^2^ |
|  | Oligo4: AAGATGCCCTCAAGGAGAAGTATTATAGTGAGTCGTATTAGGATCC^2^ |
| **2801-2821^1^** | Oligo1: GGATCCTAATACGACTCACTATAGACAGGATCCTACCACGAATA^2^ |
|  | Oligo2: AATATTCGTGGTAGGATCCTGTCTATAGTGAGTCGTATTAGGATCC^2^ |
|  | Oligo3: GGATCCTAATACGACTCACTATATATTCGTGGTAGGATCCTGTC^2^ |
|  | Oligo4:AAGACAGGATCCTACCACGAATATATAGTGAGTCGTATTAGGATCC^2^ |
| **2844-2864^1^** | Oligo1: GGATCCTAATACGACTCACTATAGAGGAAGCTCTCAGGGCTCAT^2^ |
|  | Oligo2: AAATGAGCCCTGAGAGCTTCCTCTATAGTGAGTCGTATTAGGATCC^2^ |
|  | Oligo3: GGATCCTAATACGACTCACTATAATGAGCCCTGAGAGCTTCCTC^2^ |
|  | Oligo4: AAGAGGAAGCTCTCAGGGCTCATTATAGTGAGTCGTATTAGGATCC^2^ |
| **Control^3^** | Oligo1: GGATCCTAATACGACTCACTATAGACCTACGCCACCAATTTCGT^2^ |
|  | Oligo2: AAACGAAATTGGTGGCGTAGGTCTATAGTGAGTCGTATTAGGATCC^2^ |
|  | Oligo3: GGATCCTAATACGACTCACTATAACGAAATTGGTGGCGTAGGTC^2^ |
|  | Oligo4: AAGACCTACGCCACCAATTTCGTTATAGTGAGTCGTATTAGGATCC ^2^ |

**^1^** The numbers are the starting and ending position of the target sites in Wnt5a mRNA (NM_009524.4 in NCBI).

**^2^** two pairs of DNA oligos are needed for for each double-stand siRNA. Oligo 2 is complementary with oligo 1 except an "AA" overhang at 5'; Oligo 4 is complementary with oligo 3 except an "AA" overhang at 5'. In each oligo, gene-specific sequences are underlined, other sequences are for recognition and binding by T7 RNA polymerase.

**^3^** Control siRNA does not target to any mRNA sequence in mouse.
