# Supplementary material for: Fam70A binds Wnt5a to regulate meiosis and quality of mouse oocytes
Source: Cell Prolif. 2020 May 11;53(6):e12825. doi: 10.1111/cpr.12825 (PMC7309945; doi:10.1111/cpr.12825)
Supplement: Supplementary file 5 — Table S3 [file CPR-53-e12825-s005.doc]

**Supplementary table 3. DNA oligos for APC siRNA production.**

| **Target Site** | **DNA templates** |
| --- | --- |
| **782-802^1^** | Oligo1: GGATCCTAATACGACTCACTATAGAGGTCATCTCAGAGCAGGCA^2^ |
|  | Oligo2: AATGCCTGCTCTGAGATGACCTCTATAGTGAGTCGTATTAGGATCC^2^ |
|  | Oligo3: GGATCCTAATACGACTCACTATATGCCTGCTCTGAGATGACCTC^2^ |
|  | Oligo4: AAGAGGTCATCTCAGAGCAGGCATATAGTGAGTCGTATTAGGATCC^2^ |
| **2511-2531^1^** | Oligo1: GGATCCTAATACGACTCACTATAGACGCCAATCGACATGATGAT^2^ |
|  | Oligo2: AAATCATCATGTCGATTGGCGTCTATAGTGAGTCGTATTAGGATCC^2^ |
|  | Oligo3: GGATCCTAATACGACTCACTATAATCATCATGTCGATTGGCGTC^2^ |
|  | Oligo4: AAGACGCCAATCGACATGATGATTATAGTGAGTCGTATTAGGATCC^2^ |
| **4345-4365^1^** | Oligo1: GGATCCTAATACGACTCACTATAGAATGGTGAGTGGCATCATAA^2^ |
|  | Oligo2: AATTATGATGCCACTCACCATTCTATAGTGAGTCGTATTAGGATCC^2^ |
|  | Oligo3: GGATCCTAATACGACTCACTATATTATGATGCCACTCACCATTC^2^ |
|  | Oligo4: AAGAATGGTGAGTGGCATCATAATATAGTGAGTCGTATTAGGATCC^2^ |
| **6790-6810^1^** | Oligo1: GGATCCTAATACGACTCACTATAGAGGCAGGACGATGATTCACA^2^ |
|  | Oligo2: AATGTGAATCATCGTCCTGCCTCTATAGTGAGTCGTATTAGGATCC^2^ |
|  | Oligo3: GGATCCTAATACGACTCACTATATGTGAATCATCGTCCTGCCTC^2^ |
|  | Oligo4: AAGAGGCAGGACGATGATTCACATATAGTGAGTCGTATTAGGATCC^2^ |
| **Control^3^** | Oligo1: GGATCCTAATACGACTCACTATAGACCTACGCCACCAATTTCGT^2^ |
|  | Oligo2: AAACGAAATTGGTGGCGTAGGTCTATAGTGAGTCGTATTAGGATCC^2^ |
|  | Oligo3: GGATCCTAATACGACTCACTATAACGAAATTGGTGGCGTAGGTC^2^ |
|  | Oligo4: AAGACCTACGCCACCAATTTCGTTATAGTGAGTCGTATTAGGATCC ^2^ |

**^1^** The numbers are the starting and ending position of the target sites in APC mRNA (NM_001360980.1 in NCBI).

**^2^** two pairs of DNA oligos are needed for for each double-stand siRNA. Oligo 2 is complementary with oligo 1 except an "AA" overhang at 5'; Oligo 4 is complementary with oligo 3 except an "AA" overhang at 5'. In each oligo, gene-specific sequences are underlined, other sequences are for recognition and binding by T7 RNA polymerase.

**^3^** Control siRNA does not target to any mRNA sequence in mouse.
